# Medical imaging in rheumatoid arthritis: A review on deep learning approach

**DOI:** 10.1515/biol-2022-0611

**Published:** 2023-07-06

**Authors:** Apoorva Parashar, Rahul Rishi, Anubha Parashar, Imad Rida

**Affiliations:** Department of Computer Science and Engineering, Maharshi Dayanand University, Rohtak, India; Department of Computer Science and Engineering, Manipal University Jaipur, India; BMBI Laboratory, University of Technology of Compiègne, 60200, Compiègne, France

**Keywords:** arthritis, rheumatoid arthritis, medical imaging, deep learning, hand rheumatoid arthritis, knee rheumatoid arthritis

## Abstract

Arthritis is a musculoskeletal disorder. Millions of people have arthritis, making it one of the most common joint disorders. Osteoarthritis (OA) and rheumatoid arthritis (RA) are the most common types of arthritis among the many different types available. Pain, stiffness, and inflammation are among the early signs of arthritis, which can progress to severe immobility at a later stage if left untreated. Although arthritis cannot be cured at any point in time, it can be managed if diagnosed and treated correctly. Clinical diagnostic and medical imaging methods are currently used to evaluate OA and RA, both debilitating conditions. This review is focused on deep learning approaches used by taking medical imaging (X-rays and magnetic resonance imaging) as input for the detection of RA.

## 1 Introduction

**Figure 1:**
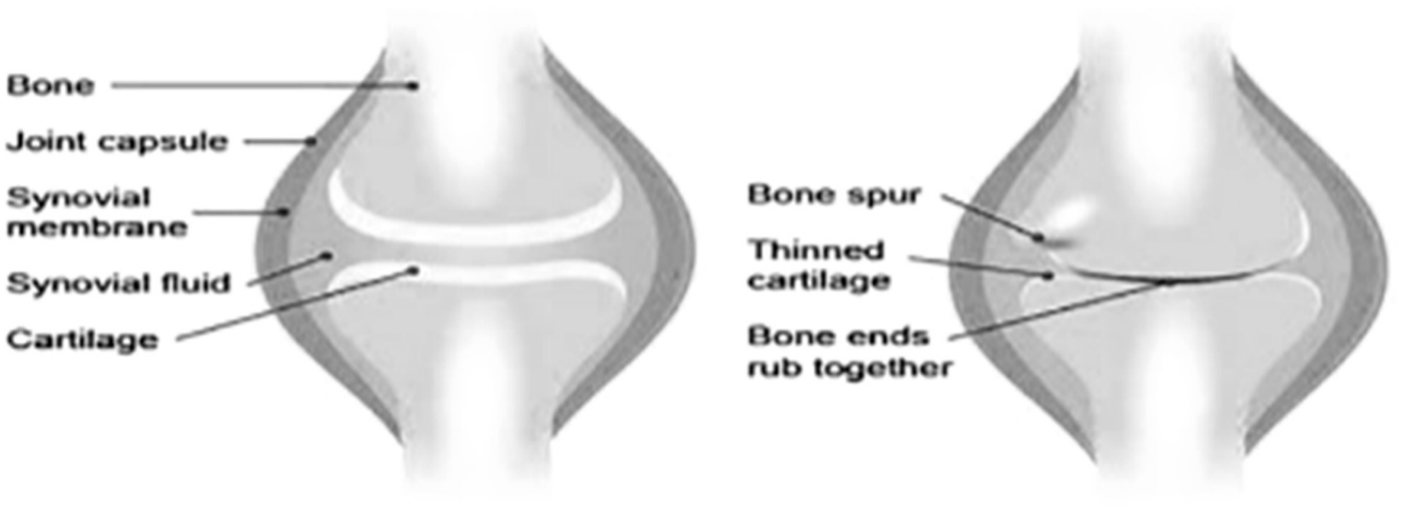
Normal knee joint and joint affected by arthritis.

Arthritis is a type of bone disease that affects the joints in the body and can be debilitating. It is most commonly found in the hand, knee, and finger joints [1]. Arthritis cannot be cured, but it can be managed at any stage of the disease. Pain, stiffness in the morning, and edema are the first signs of the disease [2–5]. If the disease is left untreated, it can produce significant immobility in the later stages of the disease. Arthritis is a disease that impacts the lives of people in numerous ways. There are several forms of arthritis, including osteoarthritis (OA) [6–13], rheumatoid arthritis (RA) [14], psoriatic arthritis, inflammatory arthritis, and others. Of these, OA and RA are the most frequent. OA can affect the ends of bones, cartilages, the femur, and other bone structures. OA is thought to be caused mainly by the degeneration of the articular cartilages (Figure 1).

The hand comprises a complex array of structures that allow for a wide range of movements, many of which are required for the performance of everyday tasks. See anatomy for further information. Wrist and forefinger synovial joints are found in many joints throughout the body, including those in hands. These joints are flexible and are surrounded by a thin, pliable membrane known as synovium, which helps to keep them flexible. Because of its ability to create synovial fluid, the synovium generally serves to nourish and lubricate the joint, allowing it to move freely. In patients who have RA, on the other hand, the joints of the hand might become inflamed when the immune system of the body malfunctions and targets healthy tissue in the fingers and wrists of the patient.

**Figure 2:**
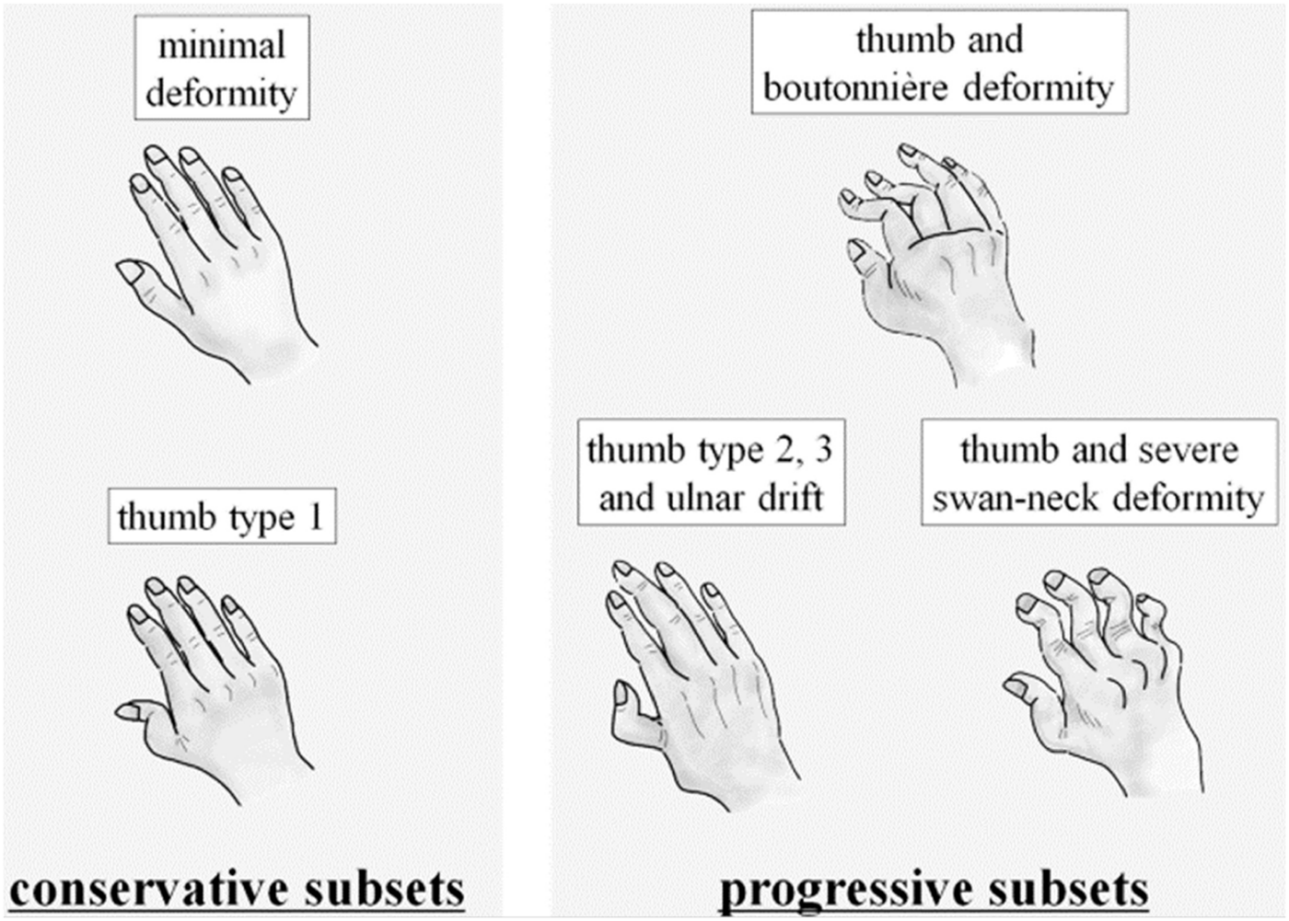
Progression of RA in hand through four stages.

There are four phases of RA: stage 1, stage 2, stage 3, and stage 4. Stage 1 is the most severe. Figure 2 depicts the progression of RA through four stages. When inflammation arises in the synovial membrane, swelling and pain occur in the joints. This is called stage 1.The number of cells in the knee region grows due to the inflammation. Synovitis is the term used to describe this stage. Pannus is the name given to the second stage of the disease, in which synovial tissue starts extend into the joint cavity, causing severe cartilage loss. Stage 3 of RA is called severe RA and is also known as fibrous ankylosis. This stage is characterized by the accumulation of synovial fluid, which results in the proliferation of synovial tissue. Because of the loss of cartilage at this time, the bone beneath the cartilage becomes visible. The last stage, known as bony ankylosis, is characterized by the cessation of joint function due to the creation of fibers or the fusing of the bone.

Current research in clinical investigation involves three types of diagnosis: medical imaging [8,15–17], blood sample analysis, and nerve conduction techniques. Medical imaging is essential in investigating arthritis in the clinical investigation because of its availability, low cost, and non-invasive nature. Blood sample analysis is also essential in investigating arthritis in clinical investigation. Medical imaging techniques that clinicians most widely utilize include X-ray imaging [8,15,18–21], ultrasound imaging [22–24], thermal imaging [16,20,25–27], and magnetic resonance imaging (MRI) [28–30], to name a few. Even though medical imaging is considered non-invasive, it does not always result in an early diagnosis. Even while the techniques for analyzing blood samples and nerve conduction are invasive, they may cause greater agony to the patients while providing little benefit in terms of correct diagnosis. Although the current standard of care for arthritis evaluation is based on medical imaging, clinical diagnosis, and symptoms, some grading systems also describe the illness as mild, moderate, or severe, depending on its severity. In medicine, the Kellgren–Lawrence (KL) [31–33] grading system is the most often used approach.

The existing approaches for clinical study of arthritis are ineffective. The best way to combat arthritis is to stop it in early stages [34] and to get diagnosed as early as possible so that disease progression can be slowed [1]. As a result, we require more precise algorithms and methodologies. In the rest of the article, Section 3 describes the methodology of the literature cited in this article. Section 2 describes the review work related to various gait distortion due to RA. Various medical imaging research has been published in various journals, covering a wide range of topics. The subjects of these investigations are diverse. The comparison is tricky because of the lack of uniformity in technique and different models. In the current study, we thoroughly analyzed RA patients who had knee and hand problems. This study examines the relationship between medical imaging techniques and RA by incorporating deep learning algorithms [5] and image processing. The majority of these studies attempt to discover effective treatments for the early identification of RA through medical imaging. When it has been shown that RA patients suffer from joint deviations, this can differentiate affected individuals from normal ones in a dataset.

### 1.1 Contribution

This systematic review included only original material that addressed medical imaging approaches tackled via deep learning in patients with RA. Some secondary literature included feet RA along with hand imaging, as some authors have worked on RA dataset for both hand and feet images. Although these approaches provide information on the early detection of RA, they do not provide apt information about which approach may be used to aid in early detection. This literature review was conducted on December 20, 2021, using four electronic databases (IEEE Xplore, PubMed, Google Scholar, and Science Direct). The following keywords were used in the search strategy (which was identical across all databases): “rheumatoid knee,” “medical imaging,” “classification,” “Deep learning approaches,” and “rheumatoid hands.” 23 articles were selected for possible addition after evaluating their titles, techniques used, and abstracts. The review included published articles in peer- reviewed conferences and journals in English. This article only had research performed on the knee joints, hand joints , and a few on the foot joints. Section 2 of the remainder of the text outlines the benchmarks that papers have utilized to detect RA. Section 3 discusses the approach used to acquire images, segment them, preprocess them, extract features, and classify them. Section 4 is a review of the research on several medical imaging modalities. Section 5 discusses the current difficulties and issues. Finally, Sections 6 and 7 consist of the conclusion and future scope.

## 2 Benchmarks

A benchmark or a point of reference is a standard against which things can be compared or evaluated. Table 1 contains the use of health assessment questionnaire (HAQ), the Sharp/van der Heijde (SvH) scoring system, KL grading system, joint space width (JSW) for OA (benchmarks), and joint used for RA detection from the literature reviewed in this article.

**Table 1:** Benchmarks and type of joints used in survey

Reference	Benchmark	Joints used
[32]	KL	Knee
[33]	KL	Knee
[35]	JSW measurement	Hand
[20]	Biochemical method HAQ	Knee
[36]	—	Hand and feet
[37]	SvH method	Hand
[38]	—	Knee
[39]	—	Knee
[40]	—	Hand
[41]	—	—
[42]	—	Knee
[43]	—	Hand
[44]	—	Hand
[45]	—	Hand
[46]	—	Hand and feet
[47]	—	Knee
[48]	SvH	Hand and feet
[49]	SvH	Hand
[15]	—	Hand
[50]	—	Hand
[51]	—	Hand
[52]	Ratingen score	Hand and feet
[53]	—	Hand

### 2.1 HAQ

Measures of health status in rheumatic diseases have gained increasing attention. Self-reported physical functioning of patients is a critical outcome measure in randomized controlled trials using questionnaires [54,55]. Clinical trials and longitudinal observational studies of RA and physical disability assessment have been suggested for daily use [56]. The Stanford Health Assessment’s inception: over two decades ago, the HAQ [57,58] facilitated the clinical evaluation of a patient’s ability to perform activities of daily life, and the HAQ has gained widespread acceptance.

The modified HAQ (MHAQ) is a condensed version of the HAQ. The MHAQ [59,60] significantly reduced the number of items in the original HAQ’s from 20 to 8 and enhanced the feasibility in clinical practice for patient screening. Both the MHAQ and the HAQ are highly responsive to clinical changes [61 however, the HAQ has proven to be more effective than the MHAQ in detecting treatment discontinuation [62]. Both the HAQ and MHAQ are beneficial in prognosis studies [63,64]. The evaluation procedures are distinct in that the HAQ includes questions about devices or assistance. This possibly increases the final score. The literature works that have used HAQ have been mentioned in Table 1.

### 2.2 SvH scoring system

Sharp’s method for scoring radiographs of the hands and feet in RA, as modified by Dr. Désirée van der Heijde (the SvH scoring method) [48,49], is now the gold standard for scoring radiographs in the majority of clinical trials and longitudinal observational studies [37]. The scoring method is largely based on Désirée van der Heijde’s article “How to read radiographs using the Sharp/van der Heijde method” in the Journal of Rheumatology, 2000; 27:261-3. The literature works that have used the SvH scoring system have been mentioned in Table 1. The SvH method includes 16 areas for erosions and 15 areas for joint space narrowing in each hand and 6 areas for erosions and joint space narrowing in each foot. It is currently the most widely used X-ray photograph scoring system in clinical trials in RA. The destruction score (DS) is based on the amount of joint surface destruction on a 0–5 scale.

### 2.3 KL grading system

The KL system is widely used for grading the severity of OA on a five-point scale. OA was graded 1 in the original paper at the following locations and projections: hands, posteroanterior cervical spine, lateral lumbar spine (only facet joints), lateral hips, anteroposterior knees, and anteroposterior feet. The following is the original description for points 0–4: Grade 0 (none): a complete absence of OA-related X-ray images; Grade 1 (doubtful): possible osteophytic lipping; Grade 2 (minimal): definite osteophytes and possible joint space narrowing; Grade 3 (moderate): numerous osteophytes, significant narrowing of joint space, some sclerosis, and possible bone end deformities; and Grade 4 (severe): numerous osteophytes, significant joint space narrowing, severe sclerosis, and definite deformity of bone ends. OA is considered present at grade 2, despite its mild severity. The literature works that have used KL grading system have been mentioned in Table 1.

### 2.4 JSW in OA

Various deep learning techniques are used to recognize gait. We provide a categorical overview of deep learning techniques along with brief descriptions of each approach used to recognize gait. The four deep learning strategies are supervised learning, unsupervised learning, hybrid learning, and reinforcement learning. There are six types of networks: feedforward, generative adversarial networks, recurrent network, hybrid learning, discriminative, and user-defined network.

Historically, OA progression has been quantified using radiographic JSW. Numerous knee radiograph protocols with varying complexities and performance have been developed to detect JSW loss (i.e., joint space narrowing). Sensitivity to joint space narrowing increases when the medial tibial plateau is radio anatomically aligned. Semiautomated software has been developed to improve the accuracy of JSW measurements over manual methods. Minimum JSW, mean JSW or joint space area, and JSW at fixed locations are all examples of JSW measurements. The literature works that have used JSW in OA are mentioned in Table 1.

## 3 Methodology

**Figure 3:**
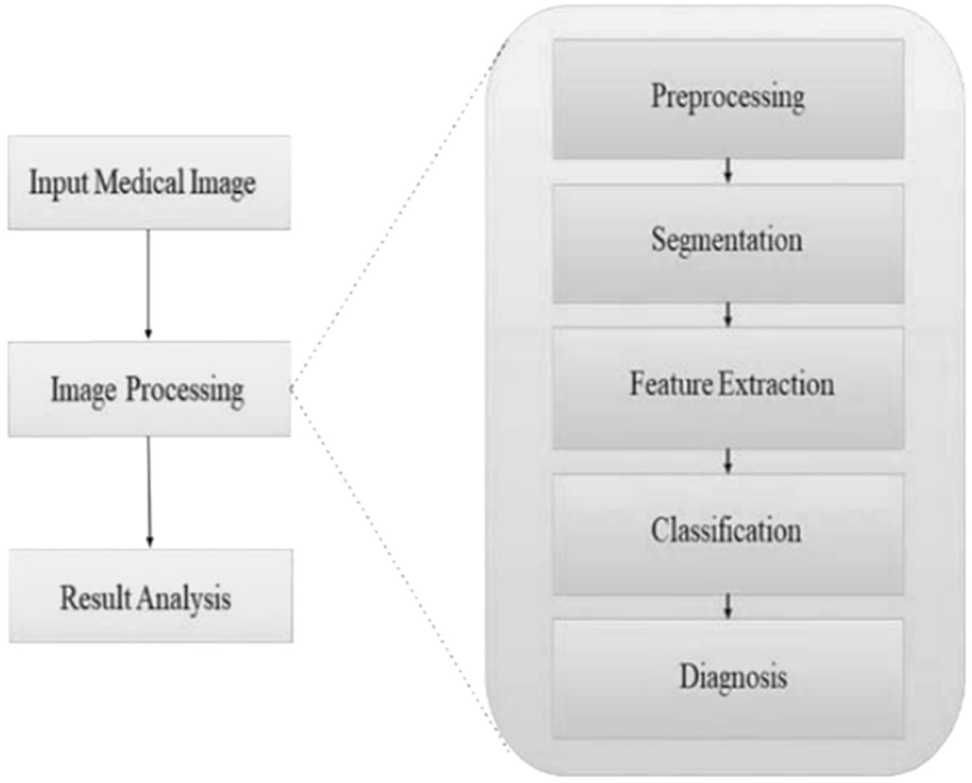
Process flow.

Any image currently being processed is subjected to the stages listed as follows: picture acquisition, preprocessing, image enhancement, segmentation, feature extraction, and classification are some techniques used. A diagram of the flow chart of the standard methodology utilized by the researchers is depicted in Figure 3.

### 3.1 Image acquisition

Papers [36,37,41,46] have acquired images from RA2 DREAM Challenge. A total of 367 patients were included in the training set for the RA2 DREAM Challenge; each patient had four photos (left/right hand/foot) used in the challenge. There were 86 target joint damage scores for each patient, 43 on each side of the body, with 21 joints narrowing and 22 erosion scores on each side of the body. On the toe joints, six toe joints were scored for both joint constriction and erosion, and they were designated as metatarsophalangeal [2,3,14,15,18].

Sample size of all the literature reviewed are mentioned in Table 2.

**Table 2:** Sample size used by various authors

Reference	Sample size
[32]	193 subjects
[33]	1,505 subjects
[35]	90 subjects
[20]	30 subjects
[36]	367 subjects
[37]	367 subjects
[38]	5 subjects
[39]	403 subjects
[40]	14 subjects
[41]	—
[42]	43 subjects
[43]	892 early RA and 1,236 non-RA subjects
[44]	132 RA subjects
[45]	91 subjects
[46]	367 subjects
[47]	—
[48]	368 subjects
[49]	108 subjects
[15]	—
[50]	30 subjects
[51]	—
[52]	—
[53]	—

### 3.2 Preprocessing

In image processing, preprocessing is a type of picture improvement that enhances some image attributes or eliminates undesirable distortions in preparation for subsequent processing. More and Singla [32] reduced the noise using a novel technique called sparse aware noise reduction utilizing convolutional neural networks (CNNs), which is described in detail below (SANR CNN). Chang et al. [33] conducted the Euclidean transformation to align the slice about a template that had been previously picked, cropped, and resized images were then used to create model training images for each knee.

The images were then selected from 11 adjacent slices on the lateral side of the center slice, 11 adjacent slices on the medial side of the center slice, and the center slice for model training for each knee. Verghese et al. [35] used Gaussian Filtering and Histogram Equalization. Snekhalatha et al. [20] performed the preprocessing of the image using an anisotropic diffusion filter, followed by a median filter to remove the background noise.

### 3.3 Image segmentation

Image segmentation is the method of partitioning a digital image into multiple segments. With the aid of a FLIR camera connected to a computer [20], thermal images of the subjects’ knees were taken on both sides of their bodies. Maziarz et al. [36] turned the joint center annotations into a segmentation mask during data preparation. Some pixels are associated with the joint center associated with the relevant joint, and others are associated with an additional background class. Yan et al. [38] used the fuzzy c-means algorithm to segment the images in this study. Based on the clusters found in the item, this segmentation method was used to divide the region of interest into subregions. The MATLAB platform is used to perform image segmentation. Dang and Allison [47] used the canny operator which operates on segmentation. They were able to locate edges by employing canning operators. This technique is the most commonly used method to detect edges and segment an image. The canny edge detector is considered to be one of the best edge detectors currently in use since it provides excellent noise immunity while still detecting actual edges.

### 3.4 Feature extraction

For image processing applications, feature extraction is the most crucial step. This stage involves computing features from the segmented images, which increases recognition accuracy when using simple classification modules. Table 3 shows different learning techniques used for extracting features. Snekhalatha [20] extracted the following statistical characteristics from the segmented X-ray image of the total population: mean value, variance, standard deviation, entropy, energy, homogeneity, sum average, difference entropy, and inverse difference moment. They were able to extract from the segmented thermal image GLCM parameters such as mean value, standard deviation, autocorrelation, dissimilarity, energy, entropy, and homogeneity for both healthy participants and RA patients. Dang and Allison [47] used pattern averaging to extract features from a dataset. It is defined as the average of the designated segments of the image, which is achieved by selecting a window of 6 × 6 segments from which the average is calculated. The literature surveyed in this article have used different learning procedures for feature extraction. The various learning types are depicted in Table 3.

**Table 3:** Learning mechanisms used by various authors

Reference	Learning used
[32]	Transfer learning
[33]	Semi-supervised learning
[35]	Supervised learning
[20]	—
[36]	Supervised learning
[37]	Supervised learning
[38]	Unsupervised learning
[39]	Supervised learning
[40]	—
[41]	Supervised learning
[42]	—
[43]	Supervised learning
[44]	Supervised learning
[45]	Supervised learning
[46]	Transfer learning
[47]	Supervised learning
[48]	Supervised learning
[49]	Supervised learning
[15]	Supervised learning
[50]	Transfer learning
[51]	Supervised learning
[52]	Transfer learning
[53]	Transfer learning

### 3.5 Classification

In this stage, the retrieved characteristics from the input photos are sent to the classifiers, which are then trained on them. The output is generated by the classifiers based on comparing the parameters with the database. To classify the outputs, the researchers employed both image processing and machine learning-based classifiers, which they combined. According to the study’s findings, some fundamental categorization approaches were applied, as shown in Table 4.

**Table 4:** Classification techniques used by various authors

References	Classifiers used
[32]	CNN
[33]	Convolutional Siamese network
[35]	—
[20]	Fast greedy snake algorithm
[36]	CNN
[37]	CNNs with attention
[38]	Fuzzy c-means algorithm
[39]	YOLO v3
[40]	Histogram smoothing
[41]	CNN
[42]	—
[43]	Multilayer CNN
[44]	K-nearest neighbors
[45]	YOLO
[46]	CNN
[47]	Artificial neural network (ANN)
[48]	CNN
[49]	CNN
[15]	CNN
[50]	CNN and ANN
[51]	CNN
[52]	CNN
[53]	YOLO v3

## 4 Systemization of RA

According to the findings of the study, researchers have investigated various approaches for the identification and analysis of RA [15,20,32,33,35–53]. Medical imaging [15,20,32,33,35–53] is critical in the diagnosis and treatment of a wide range of disorders. X-ray radiography [20,35–40,43], MRI [32,33,42], ultrasound imaging [41], and thermal imaging [20] are some of the imaging technologies that the researchers have used. Medical image processing techniques such as artificial intelligence (AI), pattern recognition, feature extraction techniques [20,47], machine learning techniques [17,24,28–31], and segmentation techniques [20,36,38,47] are all commonly used in the processing of medical images. AI, pattern recognition, and feature extraction techniques are all commonly used to process medical images.

A variety of medical imaging techniques are used to deter mine the appropriate course and severity of the disease. These include X-ray imaging, thermal imaging, ultrasound imaging, and MRI. Imaging the interior of a body for clinical examination and medical intervention and a visual representation of the function of particular organs or tissues is the technique and process known as medical imaging (physiology). It is the goal of medical imaging to expose interior structures that are concealed beneath the skin and bones, as well as to detect and cure diseases. Medical imaging also helps to build a normal anatomy and physiology database, which makes it easier to spot problems later in the process. Even though imaging of excised organs and tissues can be conducted for medical purposes, such operations are typically considered part of pathology rather than part of medical imaging. Different medical imaging techniques used by authors are mentioned in Table 5.

**Table 5:** Medical imaging techniques used by various authors

References	Type of medical imaging
[32]	MRI
[33]	MRI
[35]	X-ray
[20]	X-ray and thermography images
[36]	X-ray
[37]	X-ray
[38]	X-ray
[39]	X-ray
[40]	X-ray scan images
[41]	Ultrasound
[42]	MRI
[43]	X-ray
[44]	—
[45]	X-ray
[46]	X-ray
[47]	X-ray
[48]	X-ray
[49]	X-ray
[15]	X-ray
[50]	X-ray
[51]	X-ray
[52]	X-ray
[53]	X-ray

### 4.1 X-Rays

An extensive study has been conducted on the rate of illness progression, as shown by radiography [65–73]. RA sequential progression models have been proposed that range from a simple linear rate of progression to an S-shaped sigmoid curve, in addition to complex mathematical functions such as first-order kinetics or a square root function of the disease duration to model the sequential progression of the disease. It is difficult to compare the results of numerous investigations on the radiographic development of RA that have been published in the literature because of three primary issues. The first point to mention is the variety of study designs that have been used, including cross-sectional studies, prospective follow-up studies of patients with varying durations of disease, and prospective follow-up studies of a cohort of patients who have presented in the early stages of the disease (as opposed to later stages of the disease). The prospective study of cohorts in the early stages of development is the study design that has the fewest issues with sample bias out of the three options.

**Figure 4:**
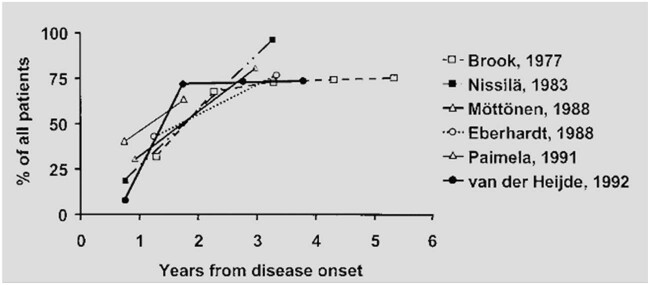
Loss function details.

The need for long-term follow-up of the entire cohort, on the other hand, presents difficulties in this regard. One of the most challenging aspects of comparing the numerous studies is the vast range of scoring systems used to measure radiographic damage [74,75]. The use of X-ray scoring systems will be described in greater detail below. From a global score for total joint damage to an objective quantitative evaluation of numerous types of abnormalities, there are various ways available for scoring. Including some form of the score for joint erosions, which stresses the relevance of joint erosions in the course of RA, is a common point of evaluation for most evaluation systems. The third challenge in conducting these comparisons is that there are so many different factors to consider, making comparative data examination difficult. Several types of data were reported, including the number of patients who had radiographic abnormalities in their joints, the number of impacted joints, the level of radiographic damage, and the course of radiographic damage, which summarize the findings of six different studies that included follow-up data. It illustrates the percentage of all patients who had joint erosions as a function of time after the onset of the disease [69]. 70% of all patients in the six prospective cohorts studied during the early stages of RA acquired erosive disease within the first year or two of disease initiation, according to the findings of the study. The literature works that have used X-rays have been mentioned in Table 5 (Figure 4).

### 4.2 MRI

When it comes to the assessment of RA, MRI is increasingly being used in both research and clinical practice because of its ability to provide insight into the pathogenesis of inflammatory joint disease and its ability to identify the key pathologic features of this disease entity at presentation much earlier than they are seen on radiography [76,77]. Both modalities are characterized by high sensitivity in depicting local inflammation in the form of synovitis, tenosynovitis, and bursitis, which are greater than that of clinical examination and conventional radiography and can aid in the establishment of an early diagnosis in RA when compared to clinical examination and conventional radiography [14].

The use of MRI can also detect bone marrow edema, which is regarded to be a precursor for the development of erosions in early RA as well as a marker of ongoing inflammation [78], and which cannot be seen using radiography, ultrasonography, or CT. Furthermore, the multiplane and multislice capabilities of MRI allow us to observe the area of interest in three orthogonal planes, which is not possible with other imaging techniques. The advantage of MRI is that it can provide information on both the bone and surrounding tissues of the joint that is not available in any other imaging method while also avoiding ionizing radiation [77]. Particularly useful in RA is MRI, which allows [79] evaluation of peripheral joints for active inflammation in the form of joint effusions, synovitis, tenosynovitis, BME, as well as subsequent structural lesions such as articular cartilage damage, cortical bone erosions, and tendon tears; and evaluation of inflammatory changes and post-inflammatory complications in the spine, including evaluation of inflammatory activity, and atlantoaxial/Atlanto-occipital. The literature works that have used MRI are mentioned in Table 5.

## 5 Ultrasound

Ultrasonography is a favored imaging technique because of its low cost, lack of exposure to hazardous radiation, and speed with which images can be acquired. Recent advancements in ultrasound image technology have enabled the development of sonographic equipment that can be used to image inflammatory joints in patients with RA, among other applications [80,81]. The literature works that have used ultrasound are mentioned in Table 5.

Image processing approaches [35,38,40,45] are being developed for the detection and categorization of RA [15,20,32,33,35–53] in various stages of development. Deep learning models [15,32,33,36,37,39,41–44,46–53] are also being used for RA detection The best appropriate technique for a specific task is of utmost importance. As a result, we may need to build a more efficient algorithm to achieve better outcomes. Researchers in arthritis detection have conducted several studies. However, it continues to be challenging to detect the existence of the disease at an early stage in the data to validate their results. Previous research [36,53] have given no information about their model pipeline. In the study by Hemalatha et al. [40], there is no information regarding the dataset used. A lot of information is vague. The weakness of such system is that there may not be a difference in the texture feature values of the normal and abnormal images. In the study by Huan et al. [42 there is no mention of methodology or how they have found the results. Dang and Allison [47] have not mentioned anything about the time taken. Use of wrong filters – Verghese et al. [35] used Gaussian filter which takes time and reduces details. Poleksic et al. [41] have used despeckling filter in which the speckle reduction depends on the diffusion coefficient and it is insufficient for the multiplicative speckle noise. Lee et al. [50] have used Sobel filter which has a lot of signal to noise ratios. They used k-fold cross validation, in which the training algorithm has to be rerun k times from scratch. There is no mention about the time taken in the study by Rohrbach et al. [51], in which Wiener filter has been used for suppressing noise, but these filters are comparatively slow to apply since they require working in the frequency domain.

There are a few more research gaps which are mentioned in Table 6. Disease progression – The most crucial step is early detection since if the disease is not detected at the appropriate stage, it will eventually result in immobility if left untreated. According to the study’s findings, only a restricted number of approaches are accessible for early detection. There are three existing approaches for detecting arthritis that has been used in clinical diagnosis over the past few years. They are imaging investigations [35,38,40,45 blood sample analyses, and nerve conduction techniques. The imaging studies are of the non-invasive variety, but even though they are non-invasive, they do not aid in diagnosing aptly at earlier stage. The other two procedures are invasive, and as a result, they may cause additional discomfort to the patients. To be sure, all the approaches listed above are ineffective for detecting arthritis in its early stages. Additionally, anatomical structures such as the tibia, cartilage, patella, bone spur, bone marrow [17,82 and others, which are very difficult to analyze, noise effects in images and multidimensional images, and other concerns have been identified by the researchers.

**Table 6:** Research gaps in the literature reviewed

References	Research gaps
[32]	Used k-fold cross validation
[33]	Their model did not show great results after checkup by physician
[35]	Used Gaussian filter
[20]	Very less sample data
[36]	Gave no information about their model pipeline
[37]	Used Adam optimizer which does not converge to an optimal solution
[38]	Very less sample data
[39]	Used k-fold cross validation
[40]	No information regarding the dataset used
[41]	Used despeckling filter
[42]	No mention of methodology or how they found the results
[43]	Used k-fold cross validation
[44]	Used Naive Bayes classifier
[45]	Used softmax activation function
[46]	Used k-fold cross validation
[47]	Did not mention time taken by their model
[48]	Implemented their model in MATLAB
[49]	They did not mention the number of epochs used for CNN
[15]	Their model did not show great results
[50]	Used Sobel filter
[51]	Used Wiener filter
[52]	Their model did not show great results
[53]	No information regarding the deep learning pipeline

### 5.1 Research gaps

The literature work that has been reviewed in this work holds some research gaps. There are a few prevalent problems in most of the papers: (1) unavailability of good datasets. Some of the authors [20,38] have used very small sample size that is insufficient to prove the actual working of their model. (2) Use of k-fold cross validation technique. k-fold cross validation [32,39,43,46] is a technique in which the training algorithm has to be rerun k times from scratch. Thus, the model would be extremely time consuming. (3) Missing data. Some literature have not given sufficient proof.

### 5.2 Potential solutions for RA

The reviewed literature on deep learning approaches for detecting RA has highlighted some research gaps that need to be addressed to improve the accuracy and effectiveness of the models. One potential solution is to ensure the availability of good datasets with sufficient sample size to validate the models. Researchers could also consider using alternative cross validation techniques that are less time-consuming than the k-fold cross validation technique. To address missing data issues, authors could provide more detailed information about their model pipeline and the datasets used. Additionally, researchers could choose appropriate filters that are efficient and effective in reducing noise without compromising on the details of the image. By addressing these research gaps, the deep learning models for detecting RA could be improved, leading to better diagnosis and treatment of the disease.

### 5.3 Discussion of the limitations of possible areas for improvement

While there are potential solutions to address the research gaps in the reviewed literature on deep learning approaches for detecting RA, there are also some limitations and possible areas for improvement. First, while increasing the sample size of datasets could improve the accuracy of the models, it may not always be possible due to the limited availability of high-quality data. Therefore, researchers could consider using data augmentation techniques to generate additional training data and improve the robustness of the models. Second, while using alternative cross validation techniques may reduce the training time of the models, it may also result in overfitting and lead to poor generalization performance. Therefore, researchers need to carefully choose the most appropriate cross validation technique that balances the trade-off between computational efficiency and generalization performance.

Third, providing more detailed information about the model pipeline and the datasets used may not always be feasible due to confidentiality and privacy concerns. However, researchers could consider providing a more detailed description of the data preprocessing steps, model architecture, and hyperparameter settings to facilitate reproducibility and transparency. Finally, while choosing appropriate filters is critical for reducing noise and preserving image details, it may also require some domain expertise and knowledge of the imaging modalities. Therefore, researchers could consider collaborating with domain experts and radiologists to choose the most appropriate filters for their specific imaging modality. Overall, while there are limitations and challenges in improving the deep learning models for detecting RA, addressing these issues could lead to more accurate and effective diagnosis and treatment of the disease.

## 6 Conclusion

A physician’s analysis of imaging tests is carried out manually, which is believed to be time-consuming and unpredictable. The presence of distortions throughout the imaging process may pose difficulties in analyzing the bone architecture. As a result, because of the problems connected with medical imaging, it is not easy to examine them quickly. Even though significant progress has been made in the development of algorithms, several challenges still need to be solved. The development of a completely automated approach or diagnostic tool for detecting arthritis at an earlier stage may be required to address these concerns.

The review conclusions are based on the findings of 23 research that met the inclusion criteria. According to the review’s findings, the significance of medical applications of knee and hand joint medical imaging data categorization and recent advancements in data gathering techniques are generating considerable interest in the issue and providing a significant impetus to the research. The review also demonstrates that biomechanical data collected during locomotion provides critical information about knee joint issues and hand joint conditions, which can be used to make an early diagnosis. When conducting further research on the issue, this survey document can be a valuable and instructive resource.

## 7 Future scope

The development of fully automated method for diagnosing arthritis will be required shortly. Researchers should improve visualization, quantify their findings, and reduce manual interactions. It is necessary to design a non-invasive type diagnosis tool in a hybrid environment that is implemented using advanced programming languages such as Python and Oracle. In order to extract more usable information from the images, it is necessary to upgrade the existing image processing and machine learning techniques. The researchers should concentrate their efforts on producing a device containing a combination of hardware and software packages within a single kit. So, an in-depth investigation into various machine learning and image processing algorithms for detecting and classifying OA and RA has been conducted with integrity.
